# The nephronophthisis-related gene *ift-139* is required for ciliogenesis in *Caenorhabditis elegans*

**DOI:** 10.1038/srep31544

**Published:** 2016-08-12

**Authors:** Shinsuke Niwa

**Affiliations:** 1 Frontier Research Institute for Interdisciplinary Sciences and Graduate School of Life Sciences, Tohoku University, Aramaki Aza Aoba 6-3, Aobaku, Sendai, Miyagi 980-8578, Japan

## Abstract

Defects in cilia cause a spectrum of diseases known as ciliopathies. Nephronophthisis, a ciliopathy, is the most common genetic cause of renal disease. Here, I cloned and analysed a nephronophthisis-related gene *ift-139* in *Caenorhabditis elegans. ift-139* was exclusively expressed in ciliated neurons in *C. elegans*. Genetic and cellular analyses suggest that *ift-139* plays a role in retrograde intraflagellar transport and is required for cilia formation. A homologous point mutation that causes ciliopathy disrupted the function of *ift-139* in *C. elegans. ift-139* is an orthologue of human *TTC21B*, mutations in which are known to cause nephronophthisis 12 and short-rib thoracic dysplasia 4. These results suggest that *ift-139* is evolutionarily conserved and fundamental to the formation of cilia.

Cilia and flagella are essential for cellular motility, sensation of extracellular stimuli and development of multicellular animals^1^,^2^. Motile cilia generate fluid flow and cell motility, while non-motile cilia work as receptors for various chemical and mechanical signalling. Though their physiological roles are different, the formation of both motile and non-motile cilia depends on a common molecular mechanism called intraflagellar transport (IFT)^1^. The IFT mechanism is very well conserved among unicellular ciliates and higher eukaryotes^1^,^3^. Disruption of IFT leads to ciliopathies including retinal degeneration, Bardet-Biedl syndrome, polycystic kidney disease, nephronophthisis and defects in the establishment of organ laterality (*situs inversus*)^1^,^4^,^5^,^6^,^7^,^8^. The cilia cytoskeleton is composed of microtubules, microtubule-associated proteins and dyneins^9^. The microtubule-dependent motors, kinesin-2 and cytoplasmic dynein-2, bind to the IFT-A and IFT-B multiprotein complexes to carry out IFT. IFT-A and IFT-B complexes are involved in the retrograde and anterograde transport of components of cilia, respectively. The molecular mechanism of IFT was first established in a unicellular ciliate *Chlamydomonas reinharditii*, where a combination of biochemistry and genetics revealed that *Chlamydomonas* IFT complex contain at least 16 proteins^10^,^11^.

*Caenorhabditis elegans (C. elegans*) is a good model for investigating the cell biology of ciliopathies^3^,^12^,^13^. Most genes associated with ciliopathies, including genes encoding IFT complex proteins, are conserved in *C. elegans* and mutations in some of these genes have been shown to lead to defects in cilia morphology and function in *C. elegans*^13^,^14^,^15^,^16^,^17^,^18^. For instance, *che-2* and *dyf-2*, originally identified as a chemotaxis mutant gene and dye-filling mutant gene, respectively encode homologues of the IFT80 and IFT144 subunits of the IFT complex^19^,^20^. Mutations in IFT80 and IFT144 genes are genetic causes of human ciliopathy^21^,^22^ and *C. elegans che-2* and *dyf-2* mutants have behavioural defects because of degenerated cilia.

Mutations in the *TTC21B* gene, which encodes the human IFT139 subunit, are causes of human ciliopathies such as NPHP12 and short-rib thoracic dysplasia 4^23^,^24^. *C. elegans* has proven to be a valuable model for the study of the function of many cilia proteins and ciliopathies. The presence of IFT139 subunit is predicted by *C. elegans* genomic analysis^25^. A promoter analysis has shown that a candidate IFT139 homologue, ZK328.7, is expressed in ciliated sensory neurons^26^. However, the function and the protein localization of *C. elegans* orthologue of *TTC21B/IFT139* has not been analyzed in detail to date. Here, I cloned and analyzed the nematode *ift-139*/*IFT139*. Moreover the effect of a mutation in *ift-139,* resulting in ciliopathy, is reported.

## Results

### *ZK328.7* is a cilia-related gene

**Figure 1:**
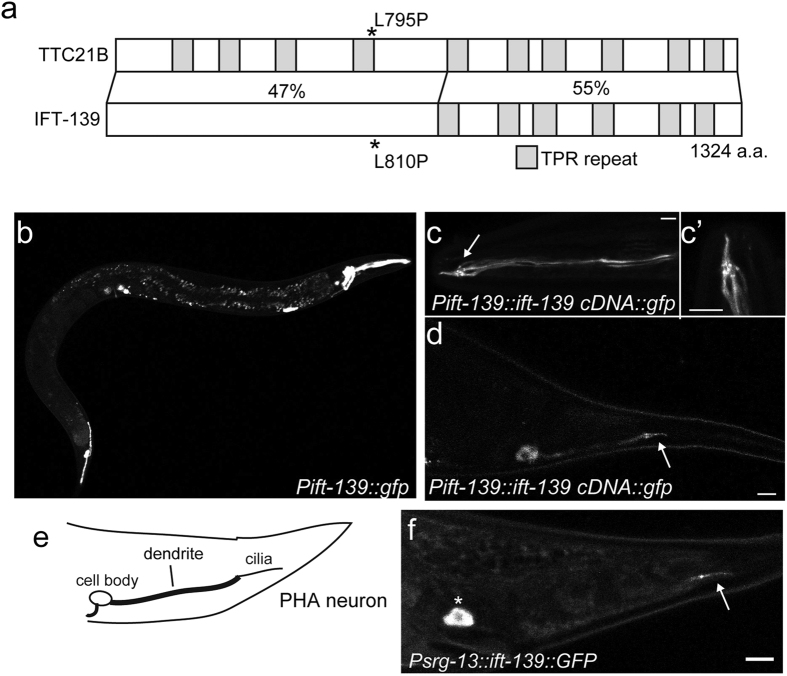
Cloning and characterisation of *ift-139.* (**a**) Comparison between TTC21B and IFT-139. Domains are predicted with the SMART algorithm. Both TTC21B and IFT-139 contain multiple TPR domains. The C-terminal halves of both proteins are predicted to have six TRP domains and the identity is 55%. While the N-terminal half of IFT-139 is not predicted to contain TPR repeats, the identity of the N-terminal half was 47%. (**b**) Expression pattern of the GFP signal under the *ift-139* promoter. Bar, 100 μm. (**c,d**) Localisation of IFT-139::GFP expressed under the *ift-139* promoter. Head neurons (**c**), amphid cilia (c’) and phasmid neurons (**d**) are illuminated. (c’) is a zoom of the cilia region in (**c**). Arrows indicate cilia. Bars, 5 μm. (**e,f**) Schematic of the PHA neuron revealed by the *srg-13* promoter (**e**) and the IFT-139::GFP signal in the PHA neuron (**f**). The asterisk and arrow indicate cell body and cilia, respectively. Bar, 5 μm.

To investigate the molecular mechanism of ciliopathy, *C. elegans* genes orthologous to human ciliopathy genes were determined. *ZK328.7* is an orthologue of the human *TTC21B* gene, mutations that cause congenital diseases such as NPHP12 and short-rib thoracic dysplasia 4 (Fig. 1a). *TTC21B* encodes the IFT139 subunit in humans^27^. *IFT139* was originally identified as a component of the IFT complex in *Chlamydomonas*^10^,^28^. Because of the homology of the protein encoded by *ZK328.7* with other IFT139 protein family members, the gene was renamed *ift-139*. Based on the predicted sequence, I performed polymerase chain reaction (PCR) from wild-type cDNA and cloned *ift-139*. While it is predicted that *ift-139* encodes two isoforms, the longer isoform which encodes a 1324-amino acid protein was obtained by 5′ UTR and 3′ UTR primer pairs. IFT-139 has at least six TPR domains, as revealed with the SMART algorithm^29^. The feature is similar to other IFT139 family members.

### IFT-139 is expressed in ciliated neurons and localises to basal bodies and cilia

To assess the cellular expression of *ift-139*, an 800-bp fragment upstream of the start codon, spanning the putative promoter region, was cloned into a Green Fluorescent Protein (GFP) expression vector and injected into wild-type nematodes. Transgenic animals were established and observed under the fluorescent microscope. GFP expression was observed in ciliated sensory neurons in both head and tail regions (Fig. 1b).

Next, the open reading frame encoding *ift-139* (1324 amino acids) was fused with GFP and expressed from the promoter described above (Fig. 1c,d). IFT-139::GFP was observed in ciliated sensory neurons. In the amphid neurons, the GFP signal was diffuse in dendrites and concentrated to the base of cilia (Fig. 1c,c’). Moreover, the cilia signal was stronger than dendrite. In phasmid PHA and PHB neurons, most of the GFP signals were observed at the base of cilia and in the cilia (Fig. 1d). Phasmid dendritic signals were relatively weak compared to amphid neurons. To observe the localisation of IFT-139 at single-cell resolution, IFT-139::GFP was expressed from the PHA neuron-specific promoter, *srg-13* promoter (Fig. 1e). Once again, the IFT-139::GFP signal was localised to the base of cilia and in the cilia of PHA neuron (Fig. 1f). These data suggest that IFT-139 may play a role in cilia.

### *ift-139* is required for the formation and function of cilia in C. elegans

**Figure 2:**
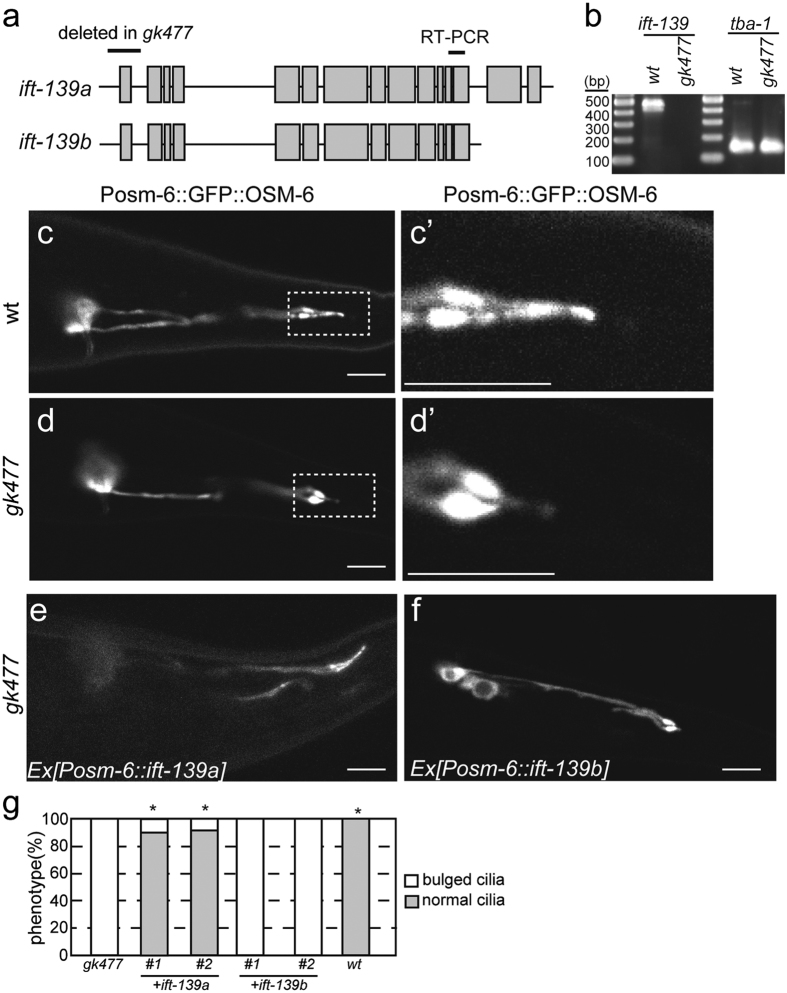
Analysis of the *ift-139* mutant. (**a**) Schematic of the *ZK328.*1/*ift-139* genomic region. Two isoforms are predicted in the database. The first exon of both isoforms is deleted in *gk477*. The region amplified in panel (**b**) is indicated. (**b**) RT-PCR was performed using primer pairs that can amplify *ift-139* and *tba-1*. Note that ift-139 is not detected in *gk477*. (**c–g**) *mnIs17[osm-6p::osm-6::gfp]* is crossed with *wt* and *ift-139(gk477)* worms and the GFP signal in PHA and PHB neurons was observed. Representative images showing the morphology of wild-type phasmid cilia (**c**,c’), *ift-139(gk477)* phasmid cilia (**d**,d’) and *ift-139(gk477)* expressing *ift-139a* cDNA (**e**) and *ift-139b* cDNA (**f**). (c’,d’) shows the boxed area in (**c**,**d**). (**g**) Graph showing the results of the rescue experiments. *p < 0.01, compared with *ift-139*. Chi-square test with Bonferroni correction. n = 40 worms.

I searched WormBase (www.wormbase.org) and found that *gk477* is a deletion mutant of *ift-139* in which the first exon of the gene is deleted (Fig. 2a). Though it is predicted that the *ift-139* genome encodes two isoforms, both isoforms share the promoter and the first exon, suggesting that both isoforms are deleted in *gk477*. To test whether *gk477* affects the expression of *ift-139*, reverse transcription polymerase chain reaction (RT-PCR) was performed using primers that can detect both isoforms (Fig. 1a). Results of RT-PCR showed that the expression of *ift-139* was under the detectable level in *gk477* (Fig. 2b), suggesting that *gk477* is a null mutant.

**Figure 3:**
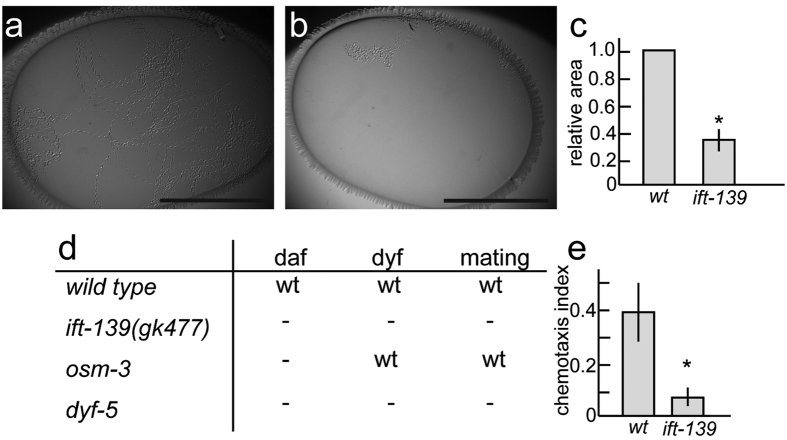
Gross phenotype of *ift-139* mutant. (**a–c**) A single worm was placed on OP50 feeder bacteria and incubated overnight. (**a,b**) Representative image of wild type (**a**) and *ift-139*(**b**). (**c**) Relative moving area was calculated. Mean ± standard deviation (S.D.). *p < 0.01, t-test, n = 10. Note that *ift-139(gk477)* mutants move in a narrow area compared with wild type. Bars, 1 cm. (**d**) Gross phenotypes in *ift-139* mutants were compared with *wild type, osm-3*, and *dyf-5* mutants. daf, dauer formation, dyf, dye filling and mating: male mating. wt means the phenotype was comparable to wt, - means the defects in the phenotype. (**e**) Chemotaxis to diacetyl was observed and chemotaxis index was calculated as described in the materials and methods. Mean ± S.D. n = 10 experiments. *p < 0.01, t-test.

Cilia morphology was investigated in nematodes carrying the *ift-139(gk477)* mutation using the well-established and widely used cilia marker, *mnIs17*^30^. This marker stably expresses OSM-6::GFP in ciliated neurons under the *osm-6* promoter. It was found that nematodes carrying the *ift-139(gk477)* mutation had abnormal cilia whose length were significantly shorter than wild type (Fig. 2c,d; wt: 6.4 ± 0.7 μm; *ift-139(gk477)*: 3.9 ± 0.7 μm; mean ± standard deviation; n = 27 cilia; p < 0.01, t-test). Cilia were also more bulged in *ift-139(gk477)* than in wild-type animals (Fig. 2d,d’). To test that this phenotype was caused by the *ift-139(gk477)* mutation, and to determine whether the effect was cell autonomous, wild-type *ift-139* cDNA was expressed specifically in ciliated neurons of the *ift-139(gk477)* mutant from the *osm-6* promoter. The activity of both long and short isoforms of *ift-139* was tested. As a result, the cilia morphology defect was rescued by expressing *ift-139a* (long isoform) but not *ift-139b* (short isoform) (Fig. 2e,f). Thus, I focused on the long isoform in this manuscript in the following experiments. To confirm that the morphological defect revealed by the GFP marker reflects abnormalities *in vivo*, phenotypes that are induced by ciliary defects were investigated. First, behaviour of *ift-139(gk477)* was observed. Single worms were placed on OP50 feeder bacteria and incubated overnight. While wild-type worms moved all over the feeder bacteria, *ift-139(gk477)* movement was restricted to a narrow area (Fig. 3a,b, 38% compared with wt). Nevertheless, unlike unc mutants, the speed of worm motility was not changed significantly when they were moving on the agarose plate. The phenotype is similar to those observed in *ift-74* and *ift-81* mutants, in which cilia are disrupted^31^. Next, dauer formation, dye-filling and mating behaviours were examined; these are known to require normal cilia function and morphology^32^,^33^,^34^. Consistent with the abnormal cilia morphology, defects in all of these processes were observed in *ift-139(gk477)* nematodes (Fig. 3d). In the *ift-139*-expressing strain (Fig. 2e), normal mating behaviour was recovered. Furthermore, previous studies have shown that *C. elegans* is attracted to diacetyl, a volatile substance, and that cilia are required for this chemotaxis^35^. We therefore calculated the chemotaxis index to diacetyl as described, comparing wild-type and *ift-139(gk477)* mutants^36^. As expected, chemotaxis was impaired, with the chemotaxis index of the *ift-139(gk477)* mutant significantly lower than wild type (Fig. 3e). Collectively, these phenotypes indicate that cilia morphology and function require *ift-139*.

### Human TTC21B can partially rescue ift-139(gk477)

**Figure 4:**
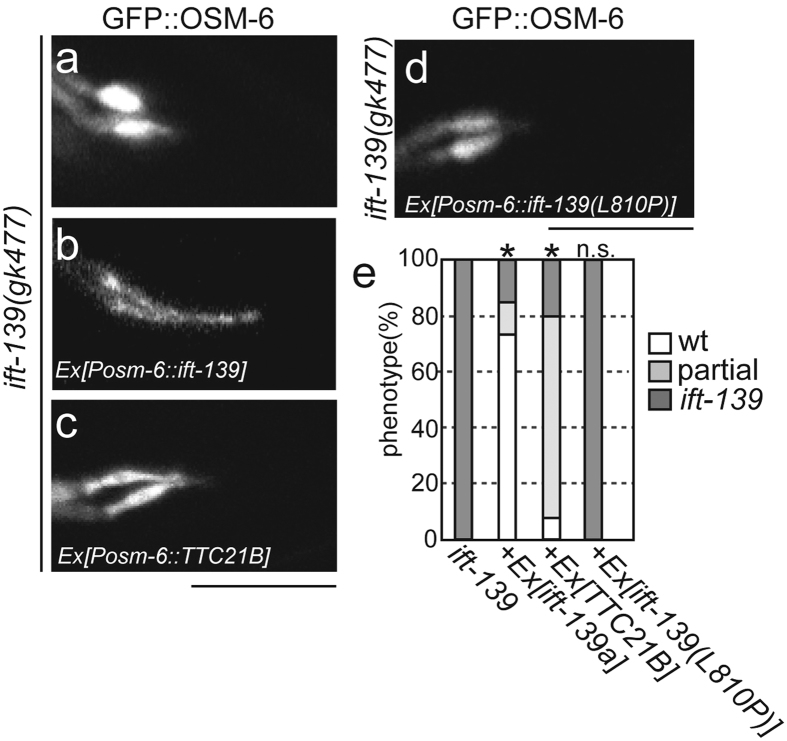
*ift-139* is related to the human *TTC21B* gene. *ift-139(gk477)*; *mnIs17[osm6p::osm-6::gfp]* (**a**) was rescued with the expression of nematode *ift-139* cDNA (**b**), human *TTC21B* (**c**) and nematode *ift-139* with the L810P mutation (**d**). Representative photographs are shown. Bars, 5 μm. (**e**) Graph showing the results of the rescue experiments. *p < 0.01, n.s, p > 0.05, compared with *ift-139*. Chi-square test with the Bonferroni correction. n = 40 worms.

*ift-139* is the nematode orthologue of *TTC21B*. To test whether the function of *ift-139* is evolutionarily conserved, the human *TTC21B* gene was cloned and fused with the *osm-6* promoter. Then, the vector was injected into *ift-139(gk477)* mutants. As a result, expressing human *TTC21B* could partially rescue cilia morphology; cilia length was longer in *ift-139(gk477)* mutants in the presence of human IFT139 than without, but shorter than wild-type cilia (Fig. 4a–c). The rescued cilia was more wild-type like morphology, but still more bulged than wild type. Statistical analysis supports the observations (Fig. 4e).

### A ciliopathy mutation disrupts the function of *ift-139*

It has been shown that *TTC21B* mutations cause autosomal recessive ciliopathies such as NPHP12 and short-rib thoracic dysplasia 4^23^,^24^,^27^. Most *TTC21B* mutations causing ciliopathies are deletions^27^, although some point mutations have been described, including a point mutation leading to an L795P substitution in human TTC21B. The L795 residue is conserved in *C. elegans* IFT-139 (L810). We postulated that a similar substitution in this conserved amino acid would result in ciliary defects in *C. elegans*. Therefore, we introduced a transgene encoding *ift-139* with the corresponding mutation, L810P, into *C. elegans ift-139(gk477)* and assessed its effect on the activity of nematode IFT-139. I tested the long isoform of *ift-139* in this experiment. While expression of wild-type *ift-139* could recover the cilia morphology of *ift-139(gk477)* mutants, expression of *ift-139(L810P)* could not (Fig. 4b,d,e). The morphology of cilia was still bulb like and could not be discriminated from *ift-139(gk477)* in IFT-139(L810P)-expressing worms. These rescue experiments by human *TTC21B* and IFT-139(L810P) suggest that the structure and function are well, but not perfectly, conserved between human TTC21B and nematode IFT-139.

### Genetic interaction suggests IFT-139 plays a role in retrograde intraflagellar transport

**Figure 5:**
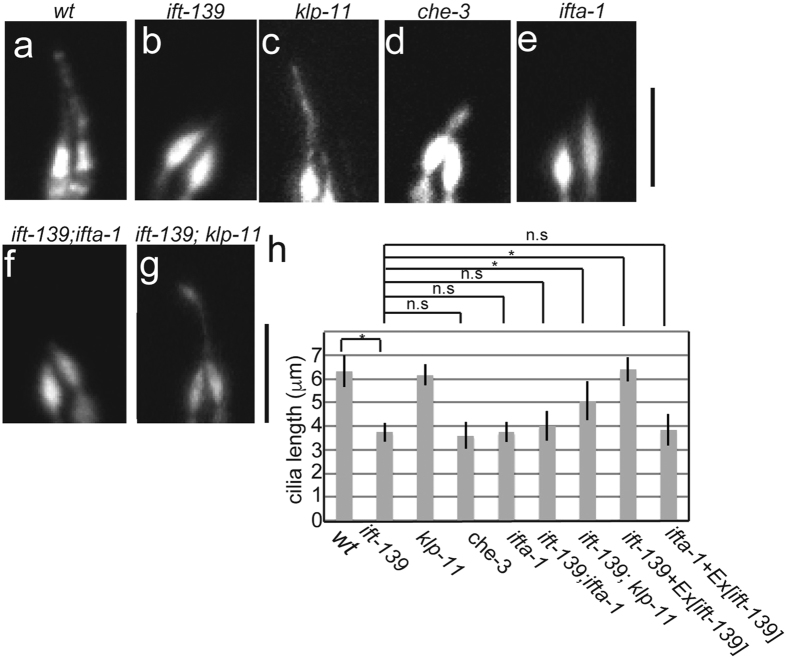
Genetic analysis of *ift-139.* Cilia morphology was observed in indicated mutants using the *mnIs17* marker. Representative images of wt (**a**), *ift-139* (**b**), *klp-11* (**c**), *che-3* (**d**), *ifta-1* (**e**), *ift-139; ifta-1* (**f**), *ift-139; klp-11* (**g**). Bars, 5 μm. (**h**) Cilia length was measured and shown as a bar graph. *p < 0.05, n.s., P > 0.05. One-way analysis of variance. n = 28.

Kinesin-2 and cytoplasmic dynein-2 anterogradely and retrogradely transport IFT particles in cilia. There are two types of kinesin-2: a heterotetramer composed of KLP-11, KLP-20 and KAP-1 and a homodimer composed of OSM-3^37^,^38^. Cytoplasmic dynein-2 is encoded by *che-3*^39^. To determine whether *ift-139* plays a role in anterograde or retrograde transport, cilia morphology was genetically analysed (Fig. 5a–h). By comparing worms carrying mutations in *ift-139* with those carrying mutations in *klp-11*, affecting anterograde transport, and *che-3*, affecting retrograde transport, it was found that *ift-139* mutations phenocopied *che-3* but not *klp-11* mutations (Fig. 5a–d). In *klp-11* mutants, while the OSM-6::GFP signal was dimmer than wild type, the length of PHA/PHB cilia was not changed significantly (Fig. 5a,c; wt: 6.3 ± 0.67 μm; *klp-11*: 6.2 ± 0.45 μm; mean ± S.D.). The phenotype was, however, clearly different from *ift-139* mutants that have shorter and bulb-like phasmid cilia (Fig. 5b; *ift-139*: 3.7 ± 0.40 μm; mean ± S.D.). In contrast, *che-3* had shorter and bulged phasmid cilia as has been previously described (Fig. 5d; *che-3*: 3.6 ± 0.55 μm; mean ± S.D.)^39^. These genetic data suggested that IFT-139 is involved in retrograde IFT. The IFT-A complex is known to contribute to retrograde IFT^40^. One component of this complex is IFT122, encoded by *ifta-1*^41^. To further confirm that *ift-139* is involved in retrograde transport, the phenotypes of *ift-139* and *ifta-1* mutants were compared. The *ifta-1* mutant had shorter and bulb-like cilia, similar to *ift-139* (Fig. 5e; *ifta-1*: 3.8 ± 0.43 μm; mean ± S.D.). Next, a double mutant was generated. Cilia morphology of the *ift-139; ifta-1* double mutant could not be discriminated from *ift-139* and *ifta-1* single mutants (Fig. 5b,e,f). The cilia length of *ift-139; ifta-1* was 4.0 ± 0.62 μm (mean ± S.D.), that is statistically identical to the *ift-139* single mutant. In contrast, an *ift-139; klp-11* double mutant had longer cilia than the *ift-139* single mutant (Fig. 5g,h; 6.4 ± 0.51 μm; mean ± S.D). In this double mutant, cilia morphology was still abnormal but more similar to wild-type rather than *ift-139* mutants (Fig. 5g). These results again suggested that *ift-139* is a component of the IFT-A complex and contributes to retrograde IFT.

### IFT-139 movement in cilia

**Figure 6:**
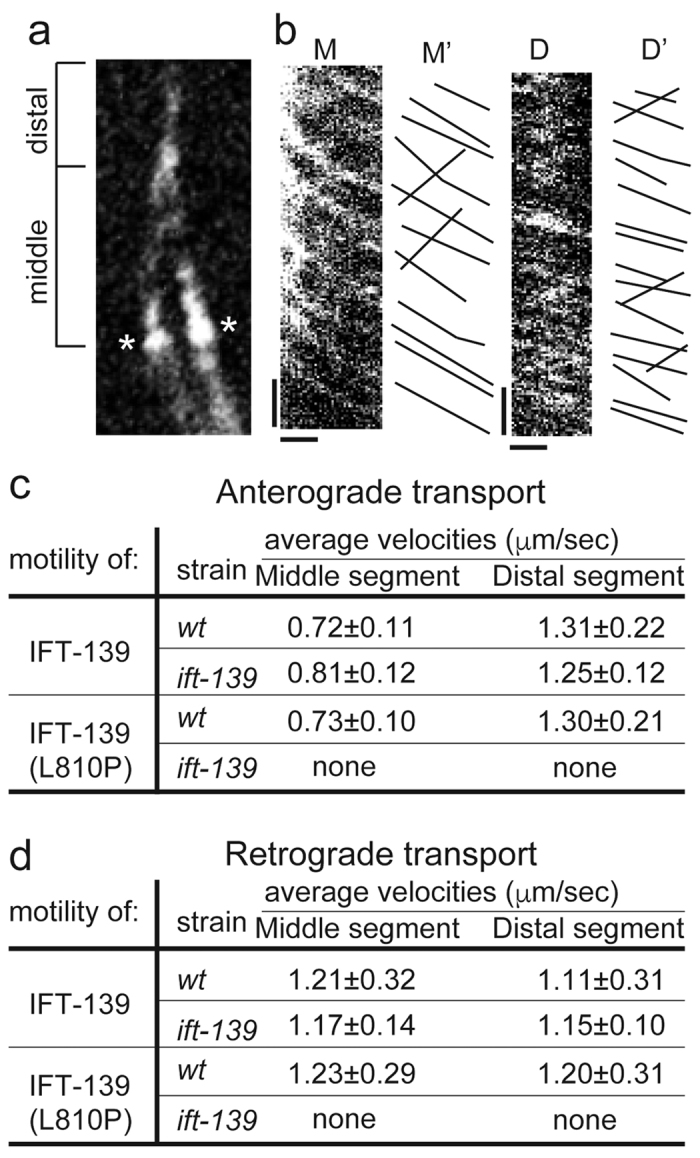
Motility of IFT139 in phasmid cilia. (**a**) Middle segment and distal segment of cilia are shown. *Indicates the transition zone. (**b**) Kymographs showing the motility of IFT-139::GFP in phasmid cilia. M and D show kymographs from the middle and distal segments, respectively. M’ and D’ show the trace from M and D. Bars, 5 s (y axis) and 1 μm (x axis). Look at supplemental movie S1. (**c**) Summary of motility parameters for the anterograde transport. As the *ift-139(gk477)* mutant expressing IFT-139(L810P) does not have normal cilia (Fig. 4d), the motility cannot be measured. n = 150 particles. Note that IFT-139::GFP moved in *wt* and *ift-139(gk477)* in a similar manner. IFT-139(L810P)::GFP moved in *wt* worms, but cannnot rescue *ift-139(gk477).* (**d**) Summary of motility parameters for the retrograde transport. n = 60 particles.

The motility of IFT-139 was observed in phasmid cilia. The long isoform of IFT-139 was expressed as a GFP fusion as described above (Fig. 1c,d) and the signal was observed by spinning disk confocal microscopy. Many IFT-139::GFP-positive particles were bidirectionally moving in the cilia (Fig. 6a,b and Supplemental Movie S1). The speed of anterograde particles was measured at 0.72 ± 0.11 μm/s and 1.31 ± 0.11 μm/s in middle and distal segments, respectively (Fig. 6a–c). The speed of retrograde transport was 1.21 ± 0.32 μm/s and 1.11 ± 0.31 μm/s in middle and distal segments, respectively (Fig. 6d). These biphasic and monophasic transport parameters in anterograde and retrograde transport are almost identical to those previously found for the IFT-particle::GFP^37^,^38^,^41^. To analyse the effect of disease-associated mutations on the motility of IFT-139, IFT-139(L810P) was fused with GFP and introduced into wild-type worms. Interestingly, in both anterograde and retrograde transport, the motility of IFT-139(L810P) was comparable to that of wild-type IFT-139 in wild-type worms (Fig. 6c,d). However, the mutant protein could not rescue *ift-139(gk477)*, suggesting that IFT-139(L810P) is normally incorporated into the IFT complex but is not functional *in vivo*.

## Discussion

The biochemistry of IFT is well studied in *Chlamydomonas*^1^,^10^,^11^, where the IFT complex is composed of at least 16 protein subunits. Most subunits are conserved among eukaryotes with cilia. The IFT139 subunit is important because mutations in *TTC21B* that encodes human IFT139 lead to ciliopathies such as NPHP12 and short-rib thoracic dysplasia 4^23^,^24^,^27^. While previous studies have identified and characterised most IFT subunits in the nematode, whether or not the *C. elegans* IFT complex contains and requires the IFT139 subunit remained uncertain. In this paper, I identified and characterised nematode *ift-139* and showed that the IFT139 subunit is essential for the formation of normal cilia. The database predicted that *ift-139* gene encodes two isoforms. However, only the long isoform (isoform a), but not the short isoform (isoform b), was essential for the intraflagellar transport (Fig. 2d–f). One possibility is that the short isoform is a prediction error. It is difficult to confirm the presence of the short isoform because of the gene structure (Fig. 2a). Another possibility is that the short isoform has roles that are different from the intraflagellar transport. Northern blotting to separate two isoforms and 3’-RACE experiments to determine the transcriptional termination would be needed to confirm the presence of the short isoform.

Genetic data suggest that IFT-139 works together with the products of *ifta-1* and *che-3. ifta-1* and *che-3* encode IFT122 and cytoplasmic dynein-2 heavy chain, both proteins that are required for retrograde IFT^39^,^41^. Thus, genetic data suggest that IFT-139 is a component of the IFT-A subcomplex in *C. elegans* and required for retrograde transport. These genetic data are consistent with the biochemical data in *Chlamydomonas* that shows that IFT139 belongs to IFT-A^42^. Interestingly, the length of cilia is significantly longer in *ift-139* mutants when a second mutation is introduced in *klp-11* (Fig. 5g). There are two possible explanations for how the *klp-11* mutation recovers cilia length in *ift-139* mutants. One possibility is that the short and bulged cilia are a result of a traffic jam induced by the *ift-139* mutation. As *klp-11* is an anterograde motor protein, loss of *klp-11* reduces the amount of anterograde transport, leading to the reduction of the traffic jam. This is supported by the observation that the amount of OSM-6::GFP is reduced in *klp-11* cilia^38^ (Fig. 5c). This idea is also consistent with the genetic data suggesting that IFT-139 is involved in retrograde transport. While it is not exclusive, another possibility is that KLP-11 transports some factors that negatively regulate cilia length^43^,^44^. This hypothesis is supported by the observation that *klp-11* mutant males have longer male-specific cilia^45^.

Disease-associated mutations often give valuable insights into the function of proteins. IFT-139(L810P) is the corresponding mutation to TTC21B(L795P) that causes ciliopathy. Previous studies in zebrafish have showed that TTC21B(L795P) is normally localised to the base of cilia^23^. We could observe the motility of this mutant protein in *C. elegans* and found that the IFT-139(L810P) protein is incorporated into the IFT particles and moves normally and bidirectionally in cilia, but cannot rescue the *ift-139* mutant (Fig. 6). Together, these results suggest that this residue is not required for the binding of IFT139 to the IFT complex, but the activity of IFT139 is disrupted by this mutation. It is possible that this residue is required to bind to a more peripheral subunit, or IFT cargo molecules such as cilia tubulin^46^. Further investigations are required to fully analyse and test these molecular interactions; however, biochemical analysis of IFT has not been established in *C. elegans*. Nevertheless, our genetic data will inform future biochemical studies in *Chlamydomonas* and mammals to reveal both how IFT139 is incorporated into, and its role in, the IFT complex.

## Methods

### Strains and Genetics

*Caenorhabditis elegans* was cultured and maintained according to the standard protocol^12^. Genotypes were determined by PCR and genomic sequencing in deletion mutants and point mutations, respectively. Primer sets used to detect mutants were as follows: *ift-139(gk477)*: 5′-GGCAGTAGCATACGAAGTGTAAGGAG-3′, 5′-CTCTCCAGTGCTCCACATCCTTGTG-3′ and 5′-GCTGTATACACCACCTAAAGCCTACC-3′. *klp-11(tm324)*: 5′-GCTCACACATTGACATAGGCCGTCG-3′, 5′-GAGACACCACTATCGGCACCAGATG-3′ and 5′-GGCTTCTTATTGTTGAAAACTTCATCCCTCG-3′. *ifta-1(gk1004*): 5′-GCACTGCTTCAACTCTTCATATGGG-3′, 5′-CCCTTCATCATCGTCATCATTTCGAATC-3′ and 5′-AGTGCCGTGCGGAACCCAGTAGATG-3′. *che-3*: 5′-TTTGCTTGGACTTGCTTGGAGCTTG-3′ and 5′-GTACTTCTGTTCAATGGATTCTTGCCTACC-3′ for PCR and 5′-TCATATTTGGGCGATCCTTTGGTCTC-3′ for sequencing. Strains are described in Supplemental Table S1.

### Construction of ift-139 promoter::gfp and cloning of ift-139 cDNA

The *ift-139* promoter, defined as the region between the last exon of the next upstream gene and the start codon of *ift-139,* was amplified from genomic DNA extracted from wild-type worms Primer sets: 5′-CTGCATGCTCCATACTCGAATACTGAG-3′ and 5′-atGGCGCGCCAAATTGAGATATCAGCAATAAAAATAT-3′. SphI and AscI sites were added to ligate the PCR product to the pSM vector. A *C. elegans* cDNA library was a generous gift from K. Mizumoto^47^. *ift-139* cDNA was amplified from the library by 2 step. Firstly, PCR was performed using 5′ UTR and 3′ UTR primer pairs and then second PCR was performed and cloned into the pSM vector using NheI and KpnI sites. PCR primers were as follows: PCR #1 was 5′-GTCAAAGCAATATTTTTATTGCTGATATCTC5′-TCACAAATGAAAGTATTCCCCATTTAC-3′ and -3′. PCR #2 was 5′-atGCTAGCaaaaATGGATTCCGAATCTGACGATAATCCAAATG-3′ and 5′-atgcGGTACCcc AGTTCTGATTAGAGCTTTCGCTTTATCCAT-3′.

### Chemotaxis assay

Well-fed adult worms were washed four times with phosphate-buffered saline. A 10-cm Petri dish containing a standard assay surface (2% agar, 5 mM K_2_PO_4_, 1 mM CaCl_2_ and 1 mM MgSO_4_) was used for the assay. Two X marks 180° opposite each other were made near the edge of the plate. 1M NaN_3_ was absorbed to these two points. After allowing the NaN_3_ to dry, approximately 50 worms were pipetted onto the plate. Quickly, 1/100 diluted diacetyl or ethanol were placed on each X mark and the lid was closed. After 1 hour, the number of worms was counted and the chemotaxis index was calculated as follows: (number of worms in diacetyl zone − number of worms in control zone)/(total number of worms).

### RT-PCR assay

Total RNA was extracted with TRIzol reagent (Thermo Fisher Scientific, MA, USA). cDNA was synthesized using SuperScript IV reverse transcriptase (Thermo Fisher Scientific) according to the manufacture’s protocol. PCR was performed using KOD FX neo DNA polymerase (TOYOBO, Tokyo, JAPAN). Primer sets are following: *ift-139*: 5′-CTCCATTTGAGCGAGATCCTGAGC-3′ and 5′-CAGCCAGAATTGAGTTAGCTTCATCG-3′. *tba-1*: 5′-GTACACTCCACTGATCTCTGCTGACAAG-3′ and 5′-CTCTGTACAAGAGGCAAACAGCCATG-3′^48^.

### Male mating assay

Males were produced by heat shock procedure. 10–15 plates that have 10 L4 worms were placed in 30 °C for 8 hours and then transferred to 20 °C. 4 days later, males were picked up. Typically, more than 20 males were obtained from all genotypes. For mating assay, 3 wt hermaphrodites were mixed with 15 males that were labelled by *mnIs17*. Mating success was judged by the presence of *mnIs17* in the F1 generation. When no F1 animals with the mnIs17 marker was found, I considered the strain exhibited defects in male mating. The phenotype was confirmed by repeating the same procedure.

### dauer-formation assay

Plates were starved for 1 week and then worms were observed using a Stemi 508 stereo microscope (Carl Zeiss, Jena, Germany). Three independent plates were prepared for each strains. Dauer was judged by the lack of pharyngeal pumping. When I could not find any dauer worms from 3 independent plates, I considered the strain dauer defective (daf). When the strain is judged as daf in the first assay, the phenotype was confirmed by repeating the same procedure.

### dye-filling assay

DiO (Thermo Fisher Scientific) was dissolved in DMSO (Sigma) at 2 mg/ml and stocked at −30 °C. Well-fed worms were collected from a 60-mm plate with 1 ml M9 buffer and pelleted at 1400 g for 2 min. Worms were resuspended in 1 ml M9 buffer and DiO was added (final 0.01 mg/ml). The mixture was slowly shaked for 3 hours. Worms were washed with M9 buffer twice. Fluorescent signal was observed with an Axiovert A1 fluorescent microscope (Carl Zeiss).

### Visualisation of cilia structure

Cilia were visualised using *mnIs17* expressing *osm-6::gfp*. OSM-6::GFP is concentrated in cilia and the base of cilia. Phasmid PHA/PHB cilia were mainly observed because the structure was easily and reproducibly detected. Amphid cilia were clouded and seemed different depending on the body angle. They were not analysed in detail. An Axiovert microscope (Carl Zeiss, Jena, Germany) equipped with a LSM710 confocal system (Carl Zeiss) and a Plan Apochromat (x63, NA1.4) objective was used to detect fluorescent signals.

### Visualisation of the IFT

Worms expressing IFT-139::GFP or IFT-139(P810L)::GFP were mounted on agarose pads and immobilised with 2 mM levamisole. Phasmid cilia were analysed on an IX81 microscope (Olympus, Tokyo, Japan) equipped with a 100× objective lens (NA1.35) and a CSU-X1 spinning disk confocal head (Yokogawa, Tokyo, Japan). The frame rate was 4 frames/s. Kymographs were made using Image J (National Institutes of Health) using the Multiple Kymograph plugin.

## Additional Information

**How to cite this article**: Niwa, S. The nephronophthisis-related gene *ift-139* is required for ciliogenesis in *Caenorhabditis elegans. Sci. Rep.* **6**, 31544; doi: 10.1038/srep31544 (2016).

## Supplementary Material

Supplementary Movie

Supplementary Information

## References

[b1] Rosenbaum J. L. & Witman G. B. Intraflagellar transport. Nat Rev Mol Cell Biol 3, 813–825, doi: 10.1038/nrm952 (2002). 12415299

[b2] Ishikawa H. & Marshall W. F. Ciliogenesis: building the cell’s antenna. Nat Rev Mol Cell Biol 12, 222–234, doi: 10.1038/nrm3085 (2011). 21427764

[b3] Scholey J. M. Cilium assembly: delivery of tubulin by kinesin-2-powered trains. Curr Biol 23, R956–R959, doi: 10.1016/j.cub.2013.09.032 (2013). 24200322

[b4] Jin H. . The conserved Bardet-Biedl syndrome proteins assemble a coat that traffics membrane proteins to cilia. Cell 141, 1208–1219, doi: 10.1016/j.cell.2010.05.015 (2010). 20603001 PMC2898735

[b5] Nachury M. V. . A core complex of BBS proteins cooperates with the GTPase Rab8 to promote ciliary membrane biogenesis. Cell 129, 1201–1213, doi: 10.1016/j.cell.2007.03.053 (2007). 17574030

[b6] Ansley S. J. . Basal body dysfunction is a likely cause of pleiotropic Bardet-Biedl syndrome. Nature 425, 628–633, doi: 10.1038/nature02030 (2003). 14520415

[b7] Nonaka S. . Randomization of left-right asymmetry due to loss of nodal cilia generating leftward flow of extraembryonic fluid in mice lacking KIF3B motor protein. Cell 95, 829–837 (1998). 9865700 10.1016/s0092-8674(00)81705-5

[b8] Mochizuki T. . PKD2, a gene for polycystic kidney disease that encodes an integral membrane protein. Science 272, 1339–1342 (1996). 8650545 10.1126/science.272.5266.1339

[b9] Kamiya R. Functional diversity of axonemal dyneins as studied in Chlamydomonas mutants. Int Rev Cytol 219, 115–155 (2002). 12211628 10.1016/s0074-7696(02)19012-7

[b10] Cole D. G. . Chlamydomonas kinesin-II-dependent intraflagellar transport (IFT): IFT particles contain proteins required for ciliary assembly in Caenorhabditis elegans sensory neurons. J Cell Biol 141, 993–1008 (1998). 9585417 10.1083/jcb.141.4.993 PMC2132775

[b11] Kozminski K. G., Beech P. L. & Rosenbaum J. L. The Chlamydomonas kinesin-like protein FLA10 is involved in motility associated with the flagellar membrane. J Cell Biol 131, 1517–1527 (1995). 8522608 10.1083/jcb.131.6.1517 PMC2120669

[b12] Brenner S. The genetics of Caenorhabditis elegans. Genetics 77, 71–94 (1974). 4366476 10.1093/genetics/77.1.71 PMC1213120

[b13] Hildebrandt F. & Zhou W. Nephronophthisis-associated ciliopathies. J Am Soc Nephrol 18, 1855–1871, doi: 10.1681/ASN.2006121344 (2007). 17513324

[b14] Cevik S. . Joubert syndrome Arl13b functions at ciliary membranes and stabilizes protein transport in Caenorhabditis elegans. J Cell Biol 188, 953–969, doi: 10.1083/jcb.200908133 (2010). 20231383 PMC2845074

[b15] Huang L. . TMEM237 is mutated in individuals with a Joubert syndrome related disorder and expands the role of the TMEM family at the ciliary transition zone. Am J Hum Genet 89, 713–730, doi: 10.1016/j.ajhg.2011.11.005 (2011). 22152675 PMC3234373

[b16] Lambacher N. J. . TMEM107 recruits ciliopathy proteins to subdomains of the ciliary transition zone and causes Joubert syndrome. Nat Cell Biol 18, 122–131, doi: 10.1038/ncb3273 (2016). 26595381 PMC5580800

[b17] Li Y., Wei Q., Zhang Y., Ling K. & Hu J. The small GTPases ARL-13 and ARL-3 coordinate intraflagellar transport and ciliogenesis. J Cell Biol 189, 1039–1051, doi: 10.1083/jcb.200912001 (2010). 20530210 PMC2886347

[b18] Warburton-Pitt S. R. . Ciliogenesis in Caenorhabditis elegans requires genetic interactions between ciliary middle segment localized NPHP-2 (inversin) and transition zone-associated proteins. J Cell Sci 125, 2592–2603, doi: 10.1242/jcs.095539 (2012). 22393243 PMC3403231

[b19] Fujiwara M., Ishihara T. & Katsura I. A novel WD40 protein, CHE-2, acts cell-autonomously in the formation of C. elegans sensory cilia. Development 126, 4839–4848 (1999). 10518500 10.1242/dev.126.21.4839

[b20] Efimenko E. . Caenorhabditis elegans DYF-2, an orthologue of human WDR19, is a component of the intraflagellar transport machinery in sensory cilia. Mol Biol Cell 17, 4801–4811, doi: 10.1091/mbc.E06-04-0260 (2006). 16957054 PMC1635379

[b21] Beales P. L. . IFT80, which encodes a conserved intraflagellar transport protein, is mutated in Jeune asphyxiating thoracic dystrophy. Nat Genet 39, 727–729, doi: 10.1038/ng2038 (2007). 17468754

[b22] Bredrup C. . Ciliopathies with skeletal anomalies and renal insufficiency due to mutations in the IFT-A gene WDR19. Am J Hum Genet 89, 634–643, doi: 10.1016/j.ajhg.2011.10.001 (2011). 22019273 PMC3213394

[b23] Davis E. E. . TTC21B contributes both causal and modifying alleles across the ciliopathy spectrum. Nat Genet 43, 189–196, doi: 10.1038/ng.756 (2011). 21258341 PMC3071301

[b24] Huynh Cong E. . A homozygous missense mutation in the ciliary gene TTC21B causes familial FSGS. J Am Soc Nephrol 25, 2435–2443, doi: 10.1681/ASN.2013101126 (2014). 24876116 PMC4214529

[b25] Cole D. G. & Snell W. J. SnapShot: Intraflagellar transport. Cell 137, 784–784.e781, doi: 10.1016/j.cell.2009.04.053 (2009). 19450523

[b26] Blacque O. E. . Functional genomics of the cilium, a sensory organelle. Curr Biol 15, 935–941, doi: 10.1016/j.cub.2005.04.059 (2005). 15916950

[b27] Tran P. V. . THM1 negatively modulates mouse sonic hedgehog signal transduction and affects retrograde intraflagellar transport in cilia. Nat Genet 40, 403–410, doi: 10.1038/ng.105 (2008). 18327258 PMC4817720

[b28] Iomini C., Li L., Esparza J. M. & Dutcher S. K. Retrograde intraflagellar transport mutants identify complex A proteins with multiple genetic interactions in Chlamydomonas reinhardtii. Genetics 183, 885–896, doi: 10.1534/genetics.109.101915 (2009). 19720863 PMC2778984

[b29] Letunic I., Doerks T. & Bork P. SMART: recent updates, new developments and status in 2015. Nucleic Acids Res 43, D257–D260, doi: 10.1093/nar/gku949 (2015). 25300481 PMC4384020

[b30] Collet J., Spike C. A., Lundquist E. A., Shaw J. E. & Herman R. K. Analysis of osm-6, a gene that affects sensory cilium structure and sensory neuron function in Caenorhabditis elegans. Genetics 148, 187–200 (1998). 9475731 10.1093/genetics/148.1.187 PMC1459801

[b31] Kobayashi T., Gengyo-Ando K., Ishihara T., Katsura I. & Mitani S. IFT-81 and IFT-74 are required for intraflagellar transport in C. elegans. Genes Cells 12, 593–602, doi: 10.1111/j.1365-2443.2007.01076.x (2007). 17535250

[b32] De Riso L., Ristoratore F., Sebastiano M. & Bazzicalupo P. Amphid defective mutant of Caenorhabditis elegans. Genetica 94, 195–202 (1994). 7896139 10.1007/BF01443433

[b33] Vowels J. J. & Thomas J. H. Genetic analysis of chemosensory control of dauer formation in Caenorhabditis elegans. Genetics 130, 105–123 (1992). 1732156 10.1093/genetics/130.1.105 PMC1204785

[b34] Barr M. M. & Sternberg P. W. A polycystic kidney-disease gene homologue required for male mating behaviour in C. elegans. Nature 401, 386–389, doi: 10.1038/43913 (1999). 10517638

[b35] Sengupta P., Chou J. H. & Bargmann C. I. odr-10 encodes a seven transmembrane domain olfactory receptor required for responses to the odorant diacetyl. Cell 84, 899–909 (1996). 8601313 10.1016/s0092-8674(00)81068-5

[b36] Ward S. Chemotaxis by the nematode Caenorhabditis elegans: identification of attractants and analysis of the response by use of mutants. Proc Natl Acad Sci USA 70, 817–821 (1973). 4351805 10.1073/pnas.70.3.817 PMC433366

[b37] Ou G., Blacque O. E., Snow J. J., Leroux M. R. & Scholey J. M. Functional coordination of intraflagellar transport motors. Nature 436, 583–587, doi: 10.1038/nature03818 (2005). 16049494

[b38] Snow J. J. . Two anterograde intraflagellar transport motors cooperate to build sensory cilia on C. elegans neurons. Nat Cell Biol 6, 1109–1113, doi: 10.1038/ncb1186 (2004). 15489852

[b39] Wicks S. R., de Vries C. J., van Luenen H. G. & Plasterk R. H. CHE-3, a cytosolic dynein heavy chain, is required for sensory cilia structure and function in Caenorhabditis elegans. Dev Biol 221, 295–307, doi: 10.1006/dbio.2000.9686 (2000). 10790327

[b40] Hao L., Efimenko E., Swoboda P. & Scholey J. M. The retrograde IFT machinery of C. elegans cilia: two IFT dynein complexes? PLoS One 6, e20995, doi: 10.1371/journal.pone.0020995 (2011). 21695221 PMC3112216

[b41] Blacque O. E. . The WD repeat-containing protein IFTA-1 is required for retrograde intraflagellar transport. Mol Biol Cell 17, 5053–5062, doi: 10.1091/mbc.E06-06-0571 (2006). 17021254 PMC1679672

[b42] Behal R. H. . Subunit interactions and organization of the Chlamydomonas reinhardtii intraflagellar transport complex A proteins. J Biol Chem 287, 11689–11703, doi: 10.1074/jbc.M111.287102 (2012). 22170070 PMC3320918

[b43] Niwa S. . KIF19A is a microtubule-depolymerizing kinesin for ciliary length control. Dev Cell 23, 1167–1175, doi: 10.1016/j.devcel.2012.10.016 (2012). 23168168

[b44] He M. . The kinesin-4 protein Kif7 regulates mammalian Hedgehog signalling by organizing the cilium tip compartment. Nat Cell Biol 16, 663–672, doi: 10.1038/ncb2988 (2014). 24952464 PMC4085576

[b45] Morsci N. S. & Barr M. M. Kinesin-3 KLP-6 regulates intraflagellar transport in male-specific cilia of Caenorhabditis elegans. Curr Biol 21, 1239–1244, doi: 10.1016/j.cub.2011.06.027 (2011). 21757353 PMC3143291

[b46] Hao L. . Intraflagellar transport delivers tubulin isotypes to sensory cilium middle and distal segments. Nat Cell Biol 13, 790–798, doi: 10.1038/ncb2268 (2011). 21642982 PMC3129367

[b47] Mizumoto K. & Shen K. Interaxonal interaction defines tiled presynaptic innervation in C. elegans. Neuron 77, 655–666, doi: 10.1016/j.neuron.2012.12.031 (2013). 23439119 PMC3846605

[b48] Hoogewijs D., Houthoofd K., Matthijssens F., Vandesompele J. & Vanfleteren J. R. Selection and validation of a set of reliable reference genes for quantitative sod gene expression analysis in C. elegans. BMC Mol Biol 9, 9, doi: 10.1186/1471-2199-9-9 (2008). 18211699 PMC2254638

