# Supplementary material for: The nephronophthisis-related gene ift-139 is required for ciliogenesis in Caenorhabditis elegans
Source: Sci Rep. 2016 Aug 12;6:31544. doi: 10.1038/srep31544 (PMC4981862; doi:10.1038/srep31544)
Supplement: Supplementary Information [file srep31544-s2.pdf]

Supplemental Information

The nephronophthisis-related gene *ift-139* is required for ciliogenesis in *Caenorhabditis elegans*

Shinsuke Niwa*

Supplemental Legends

Supplemental Movie S1 The movement of IFT-139::GFP

This movie shows the motility of IFT-139::GFP in phasmid cilia. *Pift-139::ift-139::gfp* vector was injected to *wild type* nematodes and the tail region was observed with spinning disk confocal microscopy at 20 °C. Frames were taken at 4 frames/sec.

Supplemental Table S1 Strain list

Table S1 Strain list

**strain designation**	**Genotype**	**comment**
N2	–	
VC40104	*ift-139(gk477)*	from CGC
SP2101	*ncl-1(e1865) unc-36(e251) III; osm-6(p811) V; mnIs17*	from CGC
OTL11	*mnIs17*	x6 outcrossed with N2
OTL60	*ift-139(gk477); mnIs17*	
OTL61	*che-3(e1124); mnIs17*	
OTL62	*ifta-1(gk1004); mnIs17*	
OTL63	*ift-139(gk477); klp-11(tm324); mnIs17*	
OTL64	*ift-139(gk477); ifta-1(gk1004); mnIs17*	
OTL67	*jpnEx34[Pift-139::ift-139::gfp, Punc-122::RFP]*	
OTL68	*jpnEx35[Pift-139::ift-139(P810L)::gfp, Punc-122::RFP]*	
OTL65	*ift-139; mnIs17; jpnEx32[Posm-6::ift-139(P810L), Punc-122::RFP]*	
OTL25	*ift-139; mnIs17; jpnEx10[Posm-6::ift-139, Punc-122::RFP]*	
RB1146	*dyf-5(ok1170)*	from CGC
PR802	*osm-3(p802)*	from CGC
CB1124	*che-3(e1124)*	from CGC
VC2158	*ifta-1(gk1004)*	from CGC
VC1228	*klp-11(tm324) IV*	from CGC
